# Non-protective immunity against tetanus in primiparous women and newborns at birth in rural and urban settings in Ibadan, Nigeria

**DOI:** 10.11604/pamj.supp.2017.27.3.11869

**Published:** 2017-06-23

**Authors:** Adebola Emmanuel Orimadegun, Bose Etaniamhe Orimadegun, Elijah Afolabi Bamgboye

**Affiliations:** 1 Institute of Child Health, University of Ibadan, Ibadan, Nigeria; 2 Departments of Chemical Pathology, University of Ibadan, Ibadan, Nigeria; 3 Epidemiology and Medical Statistics, College of Medicine, University of Ibadan, Ibadan, Nigeria

**Keywords:** Non-protective immunity, neonatal tetanus, tetanos quick stick, primiparous women

## Abstract

**Introduction:**

Nigeria remains among the few countries that are yet to achieve eradication of neonatal tetanus in the world despite the availability of an effective vaccine. This study investigated immunity against tetanus in primiparous mothers and neonates at birth, and identified associated factors.

**Methods:**

This cross-sectional study involved consecutive selection of 244 primiparous mother-neonate pairs (119 from rural areas, 125 from urban areas, 137 male neonates and 107 female neonates) delivered at primary healthcare facilities in Ibadan, Nigeria**.** Socio-demographic characteristics, obstetric history, immunisation and birthweight were obtained from mothers by interview. A validated immunochromatographic rapid diagnostic test kit was used to test for immunity against tetanus. Positive and negative results were interpreted as protective immunity against tetanus (PIaT) and non-protective immunity against tetanus (NPIaT), respectively. Data were analysed using descriptive statistics, Chi-square and logistic regression at p = 0.05.

**Results:**

The mean age of mothers was 27.9±3.4 years (range: 20-33) and median birthweight was 2700g (range: 1760-3300). Of the 244 mothers, 198 (81.1%) received at least two doses of tetanus toxoid injection during pregnancy and prevalence of NPIaT and PIaT was 28.7% and 71.3%, respectively. The prevalence of PIaT was significantly higher among mothers in urban areas (n= 96; 80.7%) than rural (n=78; 62.4%), p<0.001.The prevalence of NPIaT among neonates was 36.5% (n= 89). Predictors of NPIaT among neonates were residence in rural LGA (OR = 2.22; 95% CI = 1.23-3.99) and maternal tetanus immunisation <2 doses (OR = 11.68; 95% CI = 4.05-21.75).

**Conclusion:**

Lack of protective immunity against tetanus among neonates of primiparous women in Ibadan is prevalent and a more conscientious enforcement of routine tetanus prevention practices is needed.

## Introduction

According to the World Health Organization (WHO), nearly 41% of all under-five child deaths occur among newborn, in the first 28 days of life and neonatal tetanus contributes significantly to these deaths [1]. In Nigeria, neonatal tetanus accounts for about 20% of these deaths [2]. The fact that neonatal tetanus is preventable prompted the called for global elimination of the tetanus in 1988 [1]. Since then, measures have been put in place in WHO member countries where tetanus was prevalent to achieve the goal of global eradication of tetanus. Though substantial progress has been made, there are countries yet to achieve the eradication of neonatal tetanus. For instance, by June 2014, Nigeria still remained among the 24 countries that were yet to reach the tetanus elimination status while remarkable progress continues to be made in many other countries with similar demographic features and challenges through tetanus elimination programme recommended by the WHO. The methods used in countries where neonatal tetanus had been successfully eliminated include active immunisation of pregnant women and this is in place in Nigeria. Yet, neonatal tetanus continues to be a public health problem. Many Nigerian women seldom receive the recommended booster doses of tetanus toxoid after the routine immunisation in infancy [2]. Only 53% of the Nigerian women had their last birth protected against neonatal tetanus [3]. Even when the recommended two or more tetanus toxoid injections are given to women during pregnancy, the level of the protection offer by the tetanus vaccine has not been adequately documented for Nigerian women. To this end, there have been reports of neonatal tetanus in about 37.5% among neonates of vaccinated mothers, with high case fatality [4]. This fact underscores the needs for investigations into factors associated with the level and nature of protection against tetanus in primiparous women in Nigeria. This research was therefore carried out to investigate the level of protection among recently delivered primiparous women and the protection offered by acquired antibodies against tetanus in their neonates. The epidemiology of the immunity against tetanus in the Nigerian neonates has been poorly understood. It is not known “what factors are associated with protective and non-protective immunity in mothers after child-birth and in neonates on the first day of life?” This information is needed as it may influence the immunisation practices and on the effectiveness of interventions implemented in order to improve the performance of the tetanus eradication campaign. This study was, therefore, carried out to answer this question and to provide updated local information on tetanus immunisation coverage among primiparous women and their newborns in Ibadan, Nigeria.

## Methods

***Design, area and settings:*** this was a cross-sectional study. Primiparous mothers and their neonates were consecutively recruited within 24 hours after childbirth from 16 Primary Healthcare Facilities (PHFs) located in Ibadan, the capital city of Oyo state, Nigeria. Ibadan has 11 local governments areas (LGAs), made up of five within the metropolis and six surrounding the metropolitan areas. Officially, the five within the metropolis are regarded as “urban LGAs” while those at the periphery of the metropolis are “rural LGAs” [5]. The PHFs are located within the communities, and they are at readily accessible locations in order to serve as the first “point-of-call” for healthcare services.

***Study population:*** this study was focussed on women who delivered their first-ever babies. During the 20-month study period, 263 live births by primiparous women were recorded and 244 (92.8%) neonates who were singleton deliveries were recruited for the study. There were 137 male neonates and 107 female neonates. Prior to the study, it was known that over two-thirds of deliveries in Ibadan take place in these centres and all socio-economic classes utilise these facilities. Written informed consent was obtained from each of the mothers. The minimum number of women required for the study at 95% level of confidence and statistical power (1-β) of 80% was calculated as 218 using the formula for estimating sample size for single proportion [6] based on assumed prevalence of 54.7% and 10% margin of error.

***Sampling techniques:*** the final sampling unit for this study were mother-baby pairs. A three-stage sampling techniques was used to select local government areas (LGAs), two primary health centres from the list of those providing maternity services in each LGA, and to recruit mothers and their neonates as they were delivered on daily basis until the day the calculated sample size was met. Women were included in the study if they were primiparous and resident within 15 kilometres from the health centre. All those who delivered on the last recruitment day were included in the sample, provided inclusion criteria were fulfilled. Women who gave family history of allergy to any form of immunoglobulin were excluded from the study.

***Data collection, measurements and definition of variables:*** the data collection period for this study lasted approximately 20 months (14 January, 2013 to 29 September, 2014). Trained research assistants (nurses and community health officers) administered the questionnaire to mothers and examined neonates within 24 hours after delivery. The various items in the questionnaire were adapted from those used for three previous studies [7–9], this included socio-demographic characteristics, questions on knowledge and tetanus vaccination history. Knowledge about tetanus was assessed on a 10-point scale, comprising statements and questions (with possible answers as true, false, or I don't know). The reliability (internal consistency) of the knowledge scale had been tested and reported previously [10]. After administration of the questionnaire, immunity against tetanus was determined for mothers and neonates using a rapid diagnostic: Tetanos Quick Stick (TQS) (Gamma, Angleur, Belgium). The TQS detected any level of anti-tetanus antibodies ≥0.1 IU/ml [7]. Neonates who tested negative to TQS on day 1 (that is NPIaT) were given human anti-tetanus serum (ATS) immunisation injection 500 IU (Vins Bioproducts Limited, India) once after a sensitivity test was carried out by injecting 0.1 ml serum in 1:10 dilution either subcutaneously and observing for half an hour for any reactions of local or general.

***Data management and analysis:*** the main outcome variables were positive and negative test result for TQS, defined as protective and non-protective tetanus immunity against tetanus, respectively at birth (for mothers and infants). Independent variables measured included: demographic variables such as age, gender, socio-economic class, and obstetric history. The family socio-economic background was determined using the classification proposed by Oyedeji [10]. Chi-square test was utilised to test the associations between the dependent and independent categorical variables. Logistic regression analyses were performed to determine independent predictors of non-protective immunity against tetanus. Level of statistical significance was set at p = 0.05 for all the analyses. All data analyses were performed using SPSS for Windows 18.0 (SPSS Inc., IL, USA).

***Ethical considerations:*** participation in the study was completely voluntary and based on informed consent. The study proposal was reviewed and approval was obtained from the Oyo State Ethical Review Committee.

## Results

***Socio-demographic characteristics of the primiparous women:*** the mean age of the women was 27.9±3.4 years (range: 20-33). Table 1 shows the socio-demographic characteristics of the women. The majority of the women (n = 220; 90.2%) belong to the Yoruba ethnic group, while others were from non-Yoruba ethnic groups. Also, 93.0% of the women were married and living with their spouses. There were no significant differences between the distributions of women in the rural LGAs compared with urban LGAs with respect to ethnic groups (p = 0.762) and marital status (p = 0.249). However, the distribution of participants according to family socioeconomic classes revealed that the most frequent socioeconomic group was the middle class in rural LGAs (79.8%) and urban LGAs (80.0%) and none of the women fell into the high class in the urban LGAs. The distributions of women in the rural LGAs compared with urban LGAs with respect to family socioeconomic classes was statistically significant (p = 0.001).

**Table 1:** Socio-demographic characteristics of 244 mothers

Characteristics	All mothers n (%)	Mothers in Urban LGAs (N = 119) n (%)	Mothers in Rural LGAs (N = 125) n (%)	P*
**Ethnic groups**				0.762
Yoruba	220 (90.2)	107 (90.8)	112 (89.6)	
Non-Yoruba	24 (9.8)	11 (9.2)	13 (10.4)	
**Marital status**				0.249
Single/Never married	17 (7.0)	6 (5.0)	11 (8.8)	
Married living with spouse	227 (93.0)	113 (95.0)	114(91.2)	
**Religious practices**				0.728
Islam	134 (54.9)	64 (53.8)	70 (56.0)	
Christianity	110 (45.1)	55 (46.2)	55 (44.0)	
**Socio-economic class**				0.001
High	11 (4.5)	11 (8.8)	0 (0.0)	
Middle	195 (79.9)	95 (80.0)	100 (79.8)	
Low	38 (15.6)	13 (11.2)	25 (20.2)	

LGAs – Local government areas, * p values for comparison between mothers in Urban LGAs and Rural LGAs

***Knowledge of neonatal tetanus among 186 primiparous women:*** Of the 244 primiparous women, 186 (75.4%) women claimed they knew about the disease called “neonatal tetanus”, while 67 (36.0%) correctly described and/or defined it. The responses of the women to the various statements posed on tetanus and immunisation against tetanus are given in Table 2. One hundred and forty-four (77.4%) of the women who claimed they knew about the tetanus agreed that “tetanus is an infectious disease caused by contamination of wound or the umbilical stumps” while 4.8% said it was not true. Eighty-three (44.6%) of the women agreed that “the agent responsible for tetanus lives in the soil, dust, and animal waste” and 25.3% did not know. Concerning the statement “tetanus is acquired through contact with the environment, not transmitted from person to person”, 35 (18.8%) of the women chose false while 102 (54.8%) did not provide an answer. Majority (59.1%) of the women did not know that tetanus results in serious, uncontrollable muscle spasms and 34.9% agreed that muscle spasms are the cause of the “lockjaw” in infants suffering from tetanus. Notably, one hundred and eighty-four (98.9%) the women who claimed they knew about neonatal tetanus disagreed with the statement, which states: “if children have a wound or umbilical stump is smelly, they should not seek medical attention”. Fifty-four (29.0%) of the women agreed with the statement: “any wound that results in a break in the skin should be cleaned with soap and running water in order to prevent tetanus” said it was true. One hundred and seven (57.5%) of the women agreed that people who were not completely immunised and have wounds should not receive a tetanus immunisation immediately. On a 10-point scale, the mean knowledge score was 5.2±2.8 (range: 1-9). However, 55.4% (n = 103) of mothers scored less than 5 out of 10 points.

**Table 2:** Responses to some statements on tetanus among 186 primiparous women

Statements	True n (%)	False n (%)	Don’t known (%)
Tetanus is an infectious disease caused by contamination of wounds or umbilical stump in newborns	144 (77.4)*	9 (4.9)	33 (17.7)
The agent responsible for tetanus is found throughout the environment, lives in soil, dust, and animal waste.	83 (44.6)*	56 (30.1)	47 (25.3)
Tetanus is acquired through contact with the environment and it is not transmitted from person to person.	49 (26.4)*	35 (18.8)	102 (54.8)
Tetanus results in serious, uncontrollable muscle spasms. For example, the jaw is “locked” by muscle spasms, causing the disease to sometimes be called “lockjaw.”	65 (35.0)*	11 (5.9)	110 (59.1)
Tetanus may develop in people who are not immunised against it or in people who have failed to maintain adequate immunity with active booster doses of vaccine.	123 (66.1)*	34 (18.3)	29 (15.6)
If children have a wound or umbilical stump is smelly, they should not seek medical attention	2 (0.1)	184 (98.9)*	0 (0.0)
If people are not immunised against tetanus or have not kept up tetanus booster shots every 10 years, any open wound is at risk of developing tetanus.	43 (23.1)*	34 (18.3)	109 (58.6)
If individuals have trouble swallowing or have muscle spasms in the facial muscles, she/he needs to visit the emergency department for treatment immediately.	184 (98.9)*	2 (0.1)	0 (0.0)
Any wound that results in a break in the skin should be cleaned with soap and running water in order to prevent tetanus	54 (29.0)*	32 (17.2)	100 (53.8)
People who are not completely immunised and have wounds should not receive a tetanus immunisation	107 (57.5)	49 (36.4)*	30 (16.1)

* Correct answers to respective statements

***Antenatal booking and immunisation status of primiparous women :*** of the 244 women, 19 (7.8%) neither booked for antenatal care in a formal health centre nor received care from health workers during pregnancy, but of these, 7 presented at the PHF during labour and 12 soon after home delivery. In all, during the index pregnancy, 22 (9.0%) and 24 (9.8%) women did not have tetanus toxoid injection and had one dose of tetanus toxoid injection, respectively. Of the 244 primiparous women, 198 (81.1%) reported that they had received at least two doses of tetanus toxoid injection during pregnancy which showed that they had “full tetanus immunisation”. However, none of the women could recollect being given tetanus toxoid injection 1 to 2 years prior to the index pregnancy. Also, of the 198 women who reported that they had received at least two doses of tetanus toxoid injection during pregnancy, 60 (30.3%) did not have any antenatal card or case note in the health facility. Antenatal care cards were reviewed for 138 (69.7%) out of 198 women and there was no documentation for 32.8% (65/198) while evidence of receiving tetanus toxoid injection was confirmed for only 36.9% (73/198) women.

***Immunity against tetanus and Odds of non-protective immunity in primiparous women :*** of the 244 women who participated in the study, 174 (71.3%) tested positive to the “Tetanos Quick Stick” test, hence had seroprevalence of protective immunity against tetanus. Further analysis showed that the prevalence of protective immunity against tetanus among women from urban LGAs (n=96; 80.7%) was significantly higher than (n=78; 62.4%) for those residing in the rural LGAs (p < 0.001). The odds of non-protective immunity against tetanus was significantly higher in women who reside in rural LGAs than urban LGAs (OR = 2.52; 95% CI = 1.41, 4.49). Of the 22 women who reported that they did not have tetanus toxoid injection during pregnancy, only one had protective immunity against tetanus. The odds of non-protective immunity against tetanus was significantly less among those who received one dose (OR = 0.06; 95% CI = 0.01, 0.09) and those who received two doses (OR = 0.02; 95% CI = 0.01, 0.14) compared with those who had no tetanus toxoid during the index pregnancy. Significant associations were found between tetanus immunity and women's awareness about tetanus (χ^2^ = 9.687, df = 1; p = 0.002) as well as their level of knowledge about tetanus (χ^2^ = 29.447, df = 1; p <0.0001) (Table 3). The odds of non-protective immunity were significantly higher among women who reported that they had never heard of the disease called tetanus than those who knew about it (OR = 2.62; 95% CI = 1.41, 4.87). Similarly, mothers who had poor knowledge scores of tetanus had a higher odds of non-protective immunity than those who had good knowledge scores (OR = 12.54; 95% CI = 4.26, 36.91). Also, in Table 3 the prevalence of tetanus immunity by socio-demographic characteristics and the odds of testing-negative to TQS (non-protection) are displayed. Significantly more women in the non-Yoruba (70.8%) ethnic group than Yoruba ethnic group (24.1%) had non-protective immunity against tetanus. The odd of non-protective immunity against tetanus among non-Yoruba mothers was significantly higher than Yoruba (OR = 7.65; 95% CI: 3.01, 19.45). However, there were no significant associations between tetanus immunity and women's marital status as well as socioeconomic class (Table 2).

**Table 3:** Immunity against tetanus among primiparous women by knowledge and socio-demographic characteristics

Characteristics	Tetanus immunity in mothers		OR (95% CI)	P
	Non-protective n %	Protective n %		
**Age group (years)**				
20-24+	5 (12.2)	36 (87.8)	1	-
25-29	23 (20.3)	91 (79.8)	1.82 (0.64, 5.16)	0.254
30-34	42 (47.2)	47 (52.8)	6.43 (2.31, 17.91)	<0.001
Awareness about tetanus*				
No	26 (44.8)	32 (55.2)	2.62 (1.41, 4.87)	0.002
Yes+	44 (23.7)	142 (76.3)	1	-
Level of knowledge**				
Poor	40 (38.8)	63 (61.2)	12.54 (4.26, 36.91)	<0.0001
Good+	4 (4.8)	79 (95.2)	1	-
**Ethnic groups**				
Non-Yoruba	17 (70.8)	7 (29.2)	7.65 (3.01, 19.45)	<0.0001
Yoruba+	53 (24.1)	167 (75.9)	1	
**Women’s marital status**				
Single/Never married	4 (23.5)	13 (76.5)	0.75 (0.24, 2.39)	0.784***
Married living with spouse+	66 (29.1)	161 (70.9)	1	
**Socioeconomic class**				
High+	3 (27.3)	8 (72.7)	1	
Middle	55 (28.2)	140 (71.8)	1.05 (0.27, 4.09)	0.081***
Low	12 (31.6)	26 (68.4)	1.23 (0.28, 5.48)	0.071***

* Have you ever heard about the disease called “tetanus”?

*** Yates Corrected Chi-square

** Assessed on a scale of 1to 10 (Poor =score ≤4, and Good =score ≥5) among those who had heard about tetanus,

+ Reference category, OR = Odds Ratio (for being negative), CI = Confidence Interval

***Characteristics of the Neonates:*** all the neonates (224) were singleton and delivered via vaginal route; 26 (10.7%) of the births took place at home and the neonates were subsequently presented at the health facilities on the first day of life while the remaining 218 (89.3%) were delivered at the primary health facilities. In all there were 137 (56.1%) male and 107 (43.9%) female neonates, giving a male to female ratio of 1.3:1. The estimated gestational ages of the neonates ranged from 33 to 42 weeks (median = 38.0 week) and 87.7% (n = 214) were delivered at term (≥37 weeks); the prevalence of prematurity (gestational age <37 weeks) being 12.3%. The birth weight of the neonates ranged from 1760 g to 3300 g (median = 2700 g) and the prevalence of low birth weight (<2500 g) was 13.9%).

***Immunity against tetanus and odds of non-protective immunity in neonates:*** of the 244 neonates tested for tetanus immunity, 155 (63.5%) tested positive to TQS test, hence seroprevalence of protective immunity against tetanus (PIaT) was 63.5% and 89 (36.5%). The prevalence and odds of non-protective immunity against tetanus by demographic characteristics and maternal immunity were as shown in Table 4. There was a significant association between tetanus immunity in the neonates and birthweight category as well as mothers' place of residence. The odds of non-protective immunity (NPIaT) was significantly lower in normal birthweight than low birthweight neonates (OR = 0.30; 95% = 0.14, 0.63). Moreover, analysis of tetanus immunity in neonates showed that protective immunity against tetanus (PIaT) among neonates of mothers from rural LGAs (56.0%) was significantly higher than 71.4% for those residing in the urban LGAs (p = 0.012) and the odds of NPIaT was significantly more in neonates of mothers from rural LGAs than urban LGAs (OR = 1.96; 95% CI = 1.15, 3.34). Conversely, there was no significant association between tetanus immunity and gender, gestational age category and family socioeconomic class.

**Table 4:** Immunity against tetanus among neonates by socio-demographic characteristics and maternal immunity

Characteristics	Tetanus immunity in Neonates on Day 1		OR (95% CI)	P
	Non-protective n (%)	Protective n (%)		
**Gender**				
Female	40 (37.4)	67 (62.6)	1.07 (0.63-1.81)	0.795
Male^+^	49(35.8)	88(64.2)	1	
**Gestational age category**				
Preterm (<37 weeks)	8 (26.7)	22 (73.3)	0.60 (0.25-1.40)	0.233
Term (≥ 37 weeks)^+^	81 (37.9)	133 (62.1)	1	
**Birth weight category (kg)**				
≥2500 (Normal)	68 (32.4)	142 (67.6)	0.30 (0.14- 0.63)	0.001
<2500 (Low)^+^	21 (61.8)	13 (38.2)	1	
**Mothers’ place of residence**				
Rural	55 (44.0)	70 (56.0)	1.96 (1.15-3.34)	0.012
Urban^+^	34 (28.6)	85 (71.4)	1	
**Socioeconomic class**				
High^+^	3 (27.3)	8 (72.7)	1	-
Middle	73 (37.4)	122 (62.6)	1.60 (0.41-6.21)	0.132*
Low	13 (34.2)	25 (65.8)	1.39 (0.31-6.13)	0.947*
**Maternal immunisation**				
No TT or one dose	34 (73.9)	12 (26.1)	7.34 (3.56-15.25)	<0.001
Two doses^+^	55 (27.8)	143 (72.2)	1	

TT: tetanus toxoid; +: Yates corrected Chi-square; +: references category TT

***Predictors of non-protective immunity against tetanus:*** the estimates for the predictor-model to identify factors that independently predict non-protective immunity against tetanus (NPIaT) among primiparous women were as shown in Table 5. Maternal age (30-34 years), lack of awareness about tetanus and being non-Yoruba were independent predictors for NPIaT among primiparous mothers. Women aged 30-34 years had increased odds of having NPIaT by 5.26 times (95% CI = 1.70 to 9.46) compared with those in the 20-24 year age group. A mother who was not aware of tetanus was 3 times more likely to have NPIaT than those who were aware (OR = 3.0; 95% CI = 1.45, 6.15). Also, mothers from the non-Yoruba ethnic group were more likely to have NPIaT than those from Yoruba tribe (OR = 8.43; 95% CI = 3.12, 12.78). However, the Nagelkerke R Square (0.631) suggests that about 63.1% of the variation in the NPIaT among mothers could be explained by this logistic model. On the other hand, in another predictor-model to identify independent predictors for non-protective immunity against tetanus (NPIaT) among neonates (Table 6), neonates of mothers from the rural LGAs had significantly higher odds of NPIaT than those from urban LGAs (OR = 2.22; 95% CI = 1.23, 3.99). Also, neonates of mothers who received one or no doses of tetanus toxoid injection during pregnancy compared with those who received 2 doses were more likely to have NPIaT (OR = 11.68, 95% CI = 4.05, 21.75). However, the Nagelkerke R Square (0.512) showed that about 51.2% of the variation in the immunity against tetanus on the first day of life was explained by this logistic model.

**Table 5:** Predictive factors for non-protective immunity against tetanus among primiparous women

Characteristics	B	S.E.	Wald	df	p	AOR (95% CI)
**Age group (years)1**	-	-	8.29	2	0.016	-
25-29	0.57	0.73	0.61	1	0.434	1.77 (0.42, 7.35)
30-34	1.67	0.58	8.25	1	0.004	5.28 (1.70, 9.46)
Awareness about tetanus (No)2	1.11	0.37	8.84	1	0.003	3.0 (1.45, 6.15)
Ethnic groups (Non-Yoruba)3	2.12	0.51	17.68	1	<0.001	8.43 (3.12, 12.78)
Place of residence (Rural)4	-0.12	0.74	0.03	1	0.874	0.89 (0.21, 3.76)

Reference category: 120-24; 2Yes; 3Yoruba 4Urban; AOR = Adjusted Odds Ratio (for being negative), CI = Confidence Interval, Wald estimates give the “importance” of the contribution of each variable in the model, Nagelkerke R Square = 0.631

**Table 6:** Predictive factors for non-protective immunity against tetanus among neonates

Characteristics	B	S.E.	Wald	df	p	AOR (95% CI)
Place of residence (Rural)1	0.79	0.30	7.06	1	0.008	2.22 (1.23, 3.99)
Birth weight category (≥2500 kg)2	0.71	0.59	1.41	1	0.235	2.03 (0.63, 6.52)
Maternal immunisation (<2 doses of TT)3	2.46	0.54	20.65	1	<0.001	11.68 (4.05, 21.75)
Constant	-2.08	0.66	10.08	1	0.001	0.13

TT: tetanus toxoid Reference category: 1Urban; 2<2500 kg; 32 or more doses of TTAOR = Adjusted Odds Ratio (for being negative), CI = Confidence Interval, Wald estimates give the “importance” of the contribution of each variable in the model, Nagelkerke R Square = 0.512; TT:

## Discussion

The results of this study revealed that the coverage for immunisation against tetanus among primiparous women seen on the first day after childbirth in Ibadan was high, even greater than stated in the most recent Nigeria Demographic Health Survey report [3]. The awareness about tetanus, good level of knowledge about tetanus, and living in the urban LGAs positively influenced detection of protective immunity against tetanus among primiparous women in the study area. Also, failure of mothers to receive two doses of tetanus toxoid during pregnancy and residence in rural local government areas were potential risk factors for non-protective immunity against tetanus among neonates of primiparous women. Overall, our findings suggest that the primiparous mothers in Ibadan, Nigeria have no sufficient immunity to transfer to their neonates during pregnancy unless they have repeat vaccination as recommended by WHO [11]. Largely, our data appear to be a true representation of the demographic characteristic of the study population as previously reported by National Population Commission [3]. The fact that the primiparous mothers, who participated in this study from rural and urban areas of Ibadan were comparable in terms of age distributions, ethnicity, marital status and religious practices set the stage for comparisons between rural and urban areas. Also, the normality observed in the distribution of the mothers with respect to age suggests that the sampling of the study participants was, at the least, fairly random and probably devoid of selection bias. However, the number of non-Yoruba women who participated in the study were fewer than Yoruba, a reflection of the fact that the study was carried out in a Yoruba-speaking area of Nigeria. In this study, that one-tenth of the neonates were delivered at home and were brought to a primary health facilities for examination and other healthcare within 24 hours confirmed the previous report that home deliveries are a common occurrence in Ibadan [12]. It is equally important to note that a large number of home deliveries are often accidental, unprepared, and take place in the absence of medical or paramedical assistance [3, 12]. That fewer mothers correctly described and defined neonatal tetanus than the number of those who claimed to know about the disease suggests that the general knowledge of the mothers about neonatal tetanus was low. This observation agrees with previous report that knowledge about tetanus can be that low even among general public in countries with high burden [13]. Our observations about level of awareness and knowledge of neonatal tetanus among primiparous mothers are similar to those made among female adolescent girls in Ibadan in an earlier study [9]. Often, health talk about tetanus is part of routine antenatal care in a typical maternity clinic in Ibadan, Nigeria, it is not clear from our study how much of such information mothers were able to recall as at the time of interview. Overall, the implication of low knowledge of neonatal tetanus among primiparous mothers in Ibadan is that mothers do not appreciate the magnitude of the problem, the risk associated with the disease and available methods for its preventions.

The tetanus immunisation coverage recorded for pregnant women in this study is still higher than the 76.6% reported for South West in the 2013 National Demographic Health Survey [3]. However, that none of the primiparous mothers who participated in this study could recollect being given tetanus toxoid injection 1-2 years prior to pregnancy calls for public health concern. Given the mean age of the mothers (27.9 years), it was unlikely that many of the mothers had had tetanus toxoid injection during adolescence as a previous study in the same Ibadan reported a very poor immunisation experiences among high school adolescent girls [7]. It was on the basis of these observations that the investigator counselled the participating mothers and advised the health worker at the primary health facilities to give tetanus toxoid injection to those who tested negative to the TQS test before they were discharged home after delivery. Furthermore, the proportion of primiparous mothers who reported that they did not receive tetanus toxoid injection during pregnancy despite booking for antenatal clearly suggests the existence of missed opportunities for tetanus vaccination during pregnancy. Though the reason for their failure to receive the injection was not further explored, the percentage of women involved is remarkable and suggests the need for further investigation. That a remarkable number of women who reported being given tetanus toxoid injection, had no antenatal card or case note calls for concern for the quality of records currently being kept at the primary health facilities in Ibadan, Nigeria. Just as stated in the 2013 National Demographic Health Survey report [3], verbal report from mothers was used to estimate tetanus immunisation uptake among our study participants. The 71.3% overall prevalence of mothers' protective immunity against tetanus was close to 69% reported by Kurtzhals et al. [14] among women of child-bearing age in Kenya but lower than approximately 100% protection reported in Tanzania [15], Portugual [16], and Vietnam [17]. A probable explanation could be that the introduction of the immunisation programme in these three countries was earlier than in Nigeria and Kenya [18]. This massive tetanus toxoid vaccination campaigns in Tanzania [15], Portugual [16], and Vietnam [17] might have influenced the observed high level of protective immunity against tetanus. The present study further demonstrated substantial disparity between the prevalence of protective immunity against tetanus among women in the local government areas designated as “urban LGAs” and “rural LGAs” in Ibadan, Nigeria. Younger age, more recent or more booster vaccinations, or more probable exposure to other pathogens that heighten immune response among rural women may all play some roles as suggested in the data. It is, however, clear from literature that women who received only three or four doses of DPT in infancy, without additional booster doses, would most likely have lost circulating antitoxins before reaching childbearing age; but they will retain the capacity to respond to a tetanus toxoid booster dose [18].

Other factors that influenced immunity against tetanus among women who participated in this study were awareness about tetanus and level of knowledge. Women who were unaware of the disease and those who had poor knowledge had higher odds of non-protective immunity against tetanus than those who were aware and had good knowledge, respectively. These findings suggest that lack of awareness and poor knowledge might negatively affected mothers' ability to appreciate the seriousness of tetanus infection and the need to seek protection. In previous study [13], many women who lived in rural and remote areas thought that vaccination caused the loss of their pregnancy. Therefore, it was not surprising that primiparous women in the present study had remarkable difficulty in understanding the need for receiving tetanus vaccinations for neonatal tetanus prevention, hence, they had negative attitude toward it. Furthermore, this study revealed that the seroprevalence of protective immunity against tetanus among the neonates of primiparous mothers in the study area on the first day of life was 63.5%. This result clearly demonstrated the lack of adequate protective tetanus antibody levels in 36.5% of neonates. There have been few studies on protective immunity against tetanus in neonates and its persistence. The observed level of protective immunity against tetanus among the neonates in this study was considerably lower than over 90% reported for newborns in Delhi, India [19], Turkey [20], and Netherlands [21]. The higher prevalence of protective immunity against tetanus in the neonates in these countries suggests higher effectiveness of maternal tetanus immunisation campaigns than obtainable in Nigeria. Pregnant women, who have not been previously immunised, need two doses of tetanus toxoid at least one month apart early in pregnancy. This will ensure almost 100% protections for the baby and the mother [1]. Moreover, it was also observed in this study that as many as 95.5% of neonates of mothers who did not received tetanus toxoid injection during pregnancy, expectedly, had non-protective immunity against tetanus. Also, substantial numbers of those whose mothers had one dose and two doses, respectively, were not protected despite the tetanus toxoid injections given their mothers. These observations can be attributed to either missed vaccinations in the mothers or other conditions that might have impeded the transfer of tetanus specific IgG antibodies from mothers to the newborns. Tetanus immunity in adults is dose dependent and the levels and duration increase with increasing number of tetanus toxoid injection doses as documented previously [22]. However, some African infants were not protected, either due to the lack of response of their mothers to immunisation or to an insufficient antibodies transplacental transport, or to the lack of immunisation of mothers [22]. On the contrary, all European children were protected, in spite of low maternal antibody levels [23].

Previous studies have, however, shown that poorly educated women have relatively inefficient transplacental antibody transfer independent of maternal age, total antibody levels, or parity [21]. Immunoglobulins are transferred through the placenta by an active process which is Fc-gamma receptor mediated. These receptors are located in the villous macrophages and endothelium of the foetal vessels [24]. At present it is thought that IgG is transferred to the foetal circulation by binding with these receptors and then through transcellular passage [24]. It might have been informative to distinguish anti-tetanus IgG subclasses in order to predict protection in the foetus especially according to levels of educational attainment among primiparous mothers. Regrettably, this was not done in this study. Two major issues limit the generalisability and interpretations of the findings. First, three out of the eleven local government areas that constitute the Ibadan were not included in the study. Since no data were collected on the characteristics of primiparous women who delivered neonates during the study period, it is practically impossible to tell whether the inclusion of those women would have altered the findings or not. Notwithstanding this limitation, there is ample evidence to suggest that the findings might not have differed because these three LGAs comprised two rural and one urban, which have similar demographic characteristics to those included in the study. Second, though attempts were made to find out about the HIV status during pregnancy, only four mothers could provide prove of being seronegative to HIV test. Therefore, it was practically impossible to assess the impact of HIV infection on the protective immunity against tetanus among the cohort of women who took part in the study. However, previous studies have alluded to the fact that HIV might be playing important roles in the transfer of antibodies against tetanus from mothers to foetus [25]. Another factor that might have adversely affected the transfer of antibody to the foetus during pregnancy is placental malaria [26]. Again, we were not able to assess mothers for placental malaria infection and it is plausible that malaria infection may have influenced maternal transfer of antibody among primiparous women who participated in our study.

## Conclusion

In this study, the coverage for immunisation against tetanus among primiparous women at childbirth in Ibadan, Nigeria was high. Being primiparous mothers at age 30-34years and not being aware of tetanus were risk factors for non-protective immunity against tetanus among primiparous mothers. Also, neonates of women resident in rural areas and those who did not receive the recommended two doses of tetanus toxoid during pregnancy had non-protective immunity against tetanus.

### What is known about this topic

- Active immunisation of pregnant women, an important method for prevention of neonatal tetanus, is practice in Nigeria. Yet, neonatal tetanus continues to be a public health problem even among those who received the vaccination.

### What this study adds

- Risk factors for lack of protection against tetanus in neonatal life include being primiparous mothers at age 30-34years, lack of awareness of tetanus, residence in rural areas and failure to receive the recommended two doses of tetanus toxoid during pregnancy;
- The study provides information on factors that have impacts on the immunisation practice and on the effectiveness of interventions currently being implemented and those that have potential for influencing performance of the tetanus eradication campaign.

## Competing interests

The authors declare no competing interests.
